# Activation of the alternative complement pathway and its relevance for sodium retention in experimental nephrotic syndrome

**DOI:** 10.1007/s00424-025-03136-x

**Published:** 2026-01-03

**Authors:** Daniel Essigke, M. Zaher Kalo, Lingsi Kong, Matthias Wörn, Mohammad-Khaled Saad, Kingsley Omage, Bernhard N. Bohnert, Andreas L. Birkenfeld, John P. Atkinson, Xiaobo Wu, Ferruh Artunc

**Affiliations:** 1 Department of Internal Medicine, Division of Diabetology, Endocrinology and Nephrology, University Hospital Tübingen, Otfried-Mueller-Str.10, Tübingen, 72076 Germany; 2 Institute of Diabetes Research and Metabolic Diseases (IDM), Helmholtz Center Munich, University Hospital Tübingen, Tübingen, Germany; 3 German Center for Diabetes Research (DZD), University Hospital Tübingen, Tübingen, Germany; 4 Division of Nephrology and Hypertension, Department of Medicine, Oregon Health and Science University, Portland, OR USA; 5 Department of Medicine, Division of Rheumatology, Washington University School of Medicine, St. Louis, MO USA

**Keywords:** Alternative complement pathway, Nephrotic syndrome, Epithelial sodium channel, Edema, Sodium retention

## Abstract

The complement component C3, factor B (FB) and factor D (FD) belong to the alternative complement pathway and have been identified in urine samples from nephrotic mice. However, it is not yet known whether these factors are involved in mediating sodium retention in nephrotic syndrome (NS). Here we used a genetic mouse model of NS based on an inducible podocin deletion (*Nphs2*^Δipod^). These mice were intercrossed with mice deficient for FB, FD or C3, yielding *Nphs2*^Δipod^*Cfb^*−/−*^, *Nphs2*^Δipod^*Cfd^*−/−*^ *or Nphs2*^Δipod^*C3^*−/−*^ mice, respectively. NS was induced after oral doxycycline treatment for 14 days. C3, FB and FD were detected in the nephrotic urine of *Nphs2*^*Δipod*^ mice as well as fragments of C3 and FB, indicating intrarenal activation of the alternative complement pathway. Lack of FB and FD had no impact on the activation of C3. Immunohistochemistry demonstrated positive C3 staining in protein casts and within the proximal tubule. Nephrotic mice of all genotypes experienced similar proteolytic activation of the epithelial sodium channel ENaC, developed sodium retention (urinary sodium concentration < 20 mM) and body weight gain. This was associated with a stimulation of proteolytic processing of epithelial sodium channel ENaC in all genotypes. In conclusion, components of the alternative complement pathway are detectable and activated in nephrotic syndrome. Mice with deletion of C3, FB or FD are not protected from proteolytic ENaC activation and sodium retention in NS.

**Supplementary information:**

The online version contains supplementary material available at 10.1007/s00424-025-03136-x.

## Introduction

Patients with acute nephrotic syndrome (NS) are characterized by heavy proteinuria, sodium retention and edema. Considerable evidence has emerged that aberrantly filtered serine proteases resulting in proteasuria mediate sodium retention in NS by proteolytically activating the epithelial sodium channel (ENaC) expressed in the distal tubule [1–5]. This concept is supported by the findings that the cleavage products of the α- and γ-subunit of ENaC were upregulated in mice with experimental NS [6] and that the treatment with the serine protease inhibitor aprotinin prevented proteolytic ENaC activation and sodium retention as did the ENaC blocker amiloride [6–9]. In a randomized control trial involving patients with acute nephrotic syndrome, amiloride was found to be similarly effective in reducing edema compared to furosemide, indicating the involvement of ENaC-mediated sodium retention in human NS [10]. Currently, the exact identity of the serine proteases essential for ENaC activation in NS remains unknown. Proteomic analysis has identified multiple serine proteases from the plasma that are excreted in the urine of humans and mice with NS [11]. To test the relevance of some of those, we have demonstrated that the genetic deletion of urokinase plasminogen activator (*Plau*), plasmin (*Plg*), plasma kallikrein (*Klkb1*), factor VII activating protease (*Habp2*) or prostasin (*Prss8*) – all of which are aprotinin-sensitive – did not protect from sodium retention in experimental NS in mice [8, 9, 12–14].

In search of other relevant serine proteases, we identified complement factor B (FB) and factor D (FD) using a proteomic approach which were highly abundant in urine samples from nephrotic humans and mice [11]. FB and FD belong to the alternative complement pathway (AP) whereby FD as a rate-limiting protease activates FB by cleavage into Ba and Bb, liberating the catalytic domain located in Bb [15]. However, cleavage of FB by FD requires a conformational change of FB that is induced by binding of FB to either the hydrolyzed form of complement factor C3(H_2_O) which is formed spontaneously (so-called tick-over) or to the cleavage product C3b. C3(H_2_O)Bb is a C3 convertase that cleaves C3 into C3a and C3b, initiating an amplification loop to enhance classical and lectin pathways whereby C3bBb is formed, acting as a permanent and powerful AP C3 convertase [16]. This gives finally way to formation of a complement factor 5 convertase (C3bBbC3b) and initiates the terminal phase of the complement cascade. Due to their high molecular weight (MW), most complement factors such as FB (86 kDa) or C3 (186 kDa) are not excreted in the urine. FD is an exception to this rule as it has a low molecular weight (25 kDa in humans) and is filtered at the glomerulus after which it is taken up and degraded by the proximal tubule [17]. In NS there is aberrant filtration of large molecular weight complement factors, leading to excretion of these factors in the urine [18]. In addition, there is evidence of the activation of the complement system in the tubule both at the C3 level representing the alternative pathway and the terminal phase [18]. In a recent study, activation of complement factors C3 and C5 was found to be mediated by aberrantly filtered plasminogen after its activation by urokinase-type plasminogen activator (uPA) [19]. However, it is not known whether the activation of the alternative pathway is involved in the development of sodium retention by mediating proteolytic ENaC activation. In this study, we studied mice deficient for complement component 3, factor B and D regarding ENaC-mediated sodium retention in a genetic mouse model of NS based on inducible podocin deletion (*Nphs2*^Δipod^^*d*^).

## Materials and methods

### Mouse studies

Mice with two floxed podocin alleles and transgenes for a tetracycline-controlled transcriptional activation of a Cre recombinase under a podocyte-specific nephrin-driven promoter were used as a model of experimental NS (B6-Nphs2^tm3.1Antc^*Tg(Nphs1-rtTA*3G)^8Jhm^*Tg(tetO-cre)^1Jaw^ or *Nphs2*^Δipod^). These mice were intercrossed with mice deficient for complement factor B (*Cfb*^*−/−*^ [20]),, complement factor D (*Cfd*^*−/−*^ [21]), and complement factor C3 (*C3*^*−/−*^ [22]), to yield *Nphs2*^Δipod^*Cfb^*−/−*^, *Nphs2*^Δipod^*Cfd^*−/−*^ or *Nphs2*^Δipod^*C3^*−/−*^ mice, respectively. All mice were on a pure C57Bl/6 background and all genotypes were born at the expected Mendelian frequency. Genotyping was performed using PCR with the conditions and primers shown in Supplemental Table 1.

Experiments were performed on 3–6-month-old *Nphs2*^Δipod^*Cfb^*−/−*^, *Nphs2*^Δipod^*Cfd^*−/−*^, *Nphs2*^Δipod^*C3^*−/−*^ and their *Nphs2*^*Δipod*^ littermates, with mice of both sexes. Mice were kept on a 12:12-h light-dark cycle and fed a standard diet (ssniff, sodium content 0.24% corresponding to 104 µmol/g, Soest, Germany) with tap water ad libitum. Induction of experimental NS by deletion of the podocin alleles was done by a 14-day treatment with doxycycline in the drinking water (2 g/L with 5% sucrose) and the end of induction treatment was designated as day 0. During the course of nephrotic syndrome, samples of spontaneously voided urine were collected by massage of the bladder in the morning between 8 and 9 am at baseline and up to 10 days. Daily food and fluid intake were monitored by weighing the food pellets and the water bottle. Different sets of mice were used to study sodium handling, amiloride-sensitive natriuresis and the course of nephrotic syndrome. Sodium balance was studied in metabolic cages for 1 day under a control diet (C1000, Altromin, Lage, Germany, sodium content 106 µmol/g) in uninduced mice and after onset of sodium retention (urinary Sodium concentration < 20 mM) between day 2 and 7 post induction. To assess ENaC activity, amiloride-sensitive natriuresis was studied before and during sodium retention on day 7 and day 8 after end of induction. To this end, mice were injected with vehicle (5 µl/g body weight [bw] injectable water, day 7) and amiloride (10 µg/g bw) on the other day (day 8) to determine urinary sodium excretion during 6 h after injection. Amiloride-sensitive natriuresis was expressed as a ratio of both values. Daily body weight, food and fluid intake were monitored by weighing the food pellets and the water bottles. Blood samples were drawn before induction and at sacrifice on day 10.

All mouse experiments were conducted according to the National Institutes of Health Guide for the Care and Use of Laboratory Animals and the German law for the welfare of animals and were approved by local authorities (Regierungspraesidium Tuebingen).

## Laboratory measurements

Urinary creatinine was measured with a colorimetric Jaffé assay (Labor + Technik, Berlin, Germany), urinary sodium and potassium concentration as well as fecal sodium content (after dissolution in nitric acid) with flame photometry (Eppendorf EFUX 5057, Hamburg, Germany). 24 h urinary sodium and potassium excretion was normalized to body weight. Plasma urea was measured enzymatically using a colorimetric assay (Labor + Technik, Berlin, Germany). Plasma sodium and potassium were measured using an IL GEM^®^ Premier 3000 blood gas analyzer (Instrumentation Laboratory, Munich, Germany).

## Western blot analyses

The expression and activation pattern of the complement factors C3, FB and FD were analyzed using Western blots of plasma and urine samples from uninduced (healthy) and nephrotic mice of all genotypes. SDS-PAGE was performed on an 8% gel with 20 µg plasma or urinary protein per lane (or maximal volume when protein < 20 µg). Western blot analysis of ENaC subunits were performed from a membrane protein preparation of kidney cortex from uninduced and nephrotic mice of all genotypes. Half of the frozen kidney per mouse was sliced, and the cortex was dissected using a scalpel. Homogenization was performed using a Dounce homogenisator in 1 mL lysis buffer containing 250 mM sucrose, 10 mM triethanolamine HCl, 1.6 mM ethanolamine and 0.5 mM EDTA at pH 7.4 (all Sigma) [23]. During all preparation steps, aprotinin (40 µg/mL) and a protease inhibitor cocktail (final concentration 0.1 x stock; cOmplete Mini, EDTA-free, Roche) was present to avoid ENaC cleavage in vitro. Homogenates were centrifuged at 1,000 g for removal of the nuclei. Subsequently, the supernatant was centrifuged at 20,000 g for 30 min at 4 °C, and the resulting pellet containing plasma membranes was resuspended and diluted to a concentration of 5 mg/L. Native samples were boiled in Laemmli buffer at 70 °C for 10 min. For analysis of γ-ENaC cleavage fragments, samples were deglycosylated using PNGaseF according to the manufacturer´s instructions (NEB, Ipswich, USA) [24, 25]. First, samples were denaturated with a glycoprotein denaturing buffer at 70 °C for 10 min. Samples were then incubated with glycobuffer, NP-40 and PNGaseF at 37 °C for 1 h. Subsequently, 20 µg of sample were loaded on an 8% (γ-ENaC) or 4–15% (α- and β-ENaC) polyacrylamide gel for electrophoresis. After transfer to nitrocellulose membranes (Amersham Protran, Cytiva), the blocked blots (by Intercept Blocking Buffer, LI-COR, Lincoln, USA), the blocked blots were incubated with the primary antibodies. Signals were detected using fluorescent secondary antibody labelled with IRDye 800CW or IRDye 680RD and a fluorescence scanner (LI-COR Odyssey, Lincoln, USA). For loading control, total protein was measured using Revert 700 Total Protein Stain (LI-COR, Lincoln, USA). Primary antibodies are provided in Supplemental Table 2, the binding site of anti-C3 and the detection of various degradation products is provided in Supplemental Fig. 1.

## Immunohistochemistry

For analysis of tissue expression of complement factor C3 and γ-ENaC, kidneys were collected under control conditions or after 8 days after induction of experimental nephrotic syndrome. Paraffin-embedded formalin-fixed Sect. (1 μm) were deparaffinized with ethanol and rehydrated using standard protocols. Antigen retrieval was accomplished after heating for 5 min in antigen retrieval solution pH 6.1 (DAKO Deutschland GmbH, Hamburg, Germany) using a pressure cooker (Rommelsbacher, Germany). Kidney sections were blocked with avidin and biotin for each 15 min, followed by blocking for another 30 min with normal goat serum diluted 1:5 in 50 mM tris(hydroxymethyl)-aminomethane (Tris), pH 7.6 and 0.1 mL Tween 20%, supplemented with 5% (w/v) skim milk (Bio-Rad Laboratories, Munich, Germany). Sections were incubated overnight at 4 °C with the primary antibodies (dilutions 1:1000 for Anti-C3 and 1:250 for Anti-γ-ENaC) and subsequent washing in Tris buffer (50 mM Tris, pH 7.4, supplemented with 0.05% (v/v) Tween 20 (Sigma-Aldrich, Munich, Germany; 3 x). A secondary antibody (biotinylated goat anti-rabbit, Vector Laboratories, Burlingame, CA, USA; 1:500) was applied for 30 min at room temperature. Sections were further processed using the VectaStain ABC kit according to the manufacturer’s instructions and DABImmPact (both Vector Laboratories) as substrate. Finally, the sections were counterstained in hematoxylin, dehydrated, and mounted for observation using a Zeiss upright microscope. For each staining, 4 sections from at least two mice were analyzed at 20x and 63x magnification to be able to make a qualitative statement.

### Statistical analysis

Data are provided as means with SEM. Data were tested for normality with the Kolmogorov-Smirnov-Test, D’Agostino and Pearson omnibus normality test and Shapiro-Wilk-Test. Variances were tested using the Bartlett´s test for equal variances. Accordingly, data were tested for significance with parametric ANOVA followed by Dunnett´s Multiple Comparison post-test or nonparametric Kruskal-Wallis followed by Dunn´s Multiple Comparison post-test, paired or unpaired Student’s t-test, or Wilcoxon-test where applicable using GraphPad Prism 10, GraphPad Software (San Diego, CA, www.graphpad.com). Densitometric analysis of the Western blots was done using Image Studio Version 3.1.4 and Empiria Studio Version 1.3.0.83 (Licor). A p value < 0.05 at two-tailed testing was considered statistically significant.

## Results

### Activation of complement component C3 in the plasma after induction of nephrotic syndrome

**Fig. 1:**
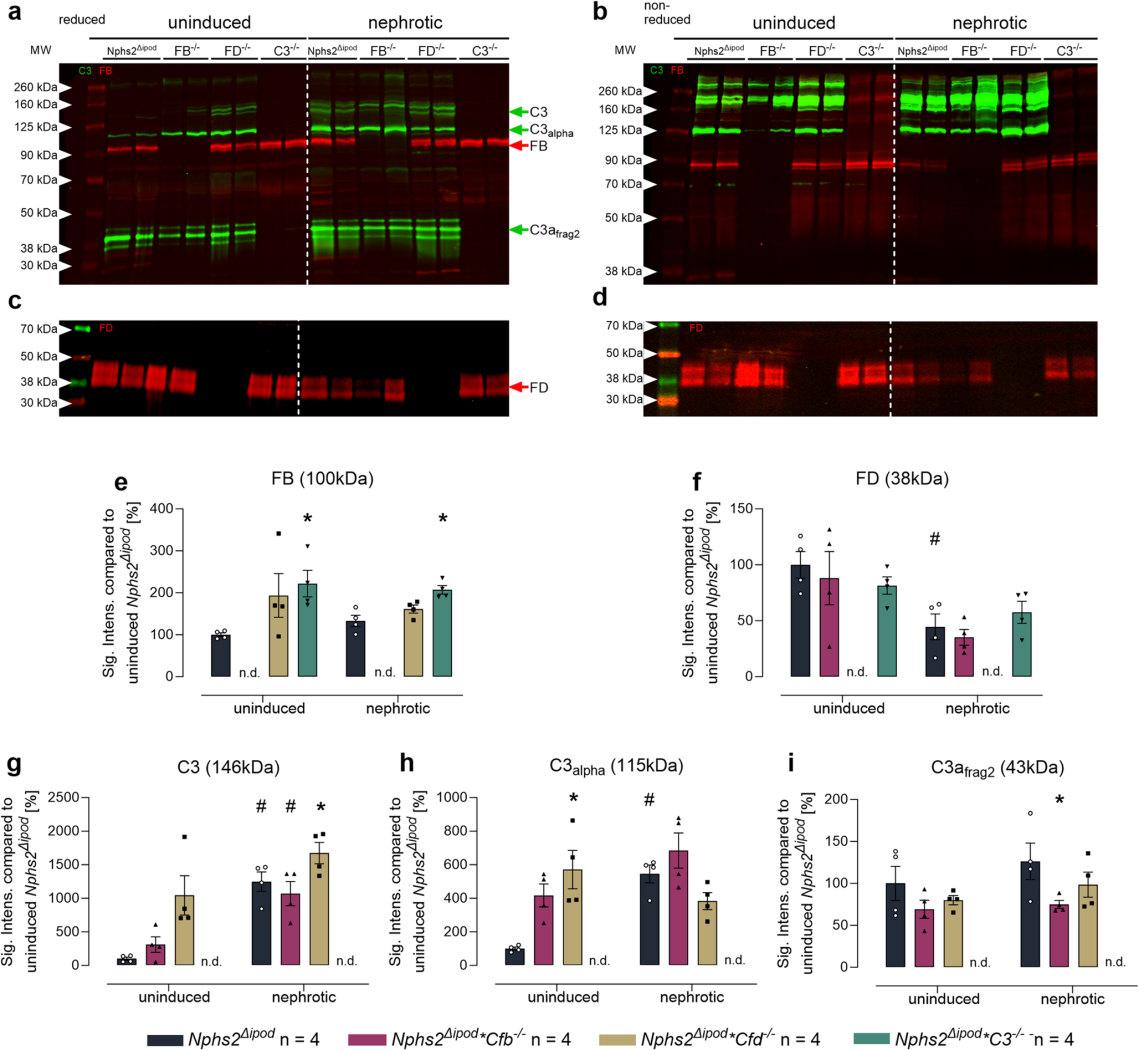
Expression of FB, FD and C3 in the plasma of *Nphs2*^Δipod^*, Nphs2*^Δipod^*Cfb^-/-^*(FB*^-/-^*), Nphs2*^Δipod^*Cfd^-/-^ (FD^-/-^) and *Nphs2*^Δipod^*C3^-/-^(C3^-/-^) mice before and after induction of experimental nephrotic syndrome. **a, b** Western blot for expression of C3 (green) and FB (red) under reducing **a** or non-reducing conditions **b**. **c, d** Western blot for expression of FD (red) under reducing **c** or non-reducing conditions **d**. **e–i** Densitometry of the obtained bands under reducing conditions. Note the specificity of the antibodies used as they lack any signal in the respective ko strain. ^#^ Significant difference (*p* < 0.05) between uninduced and nephrotic mice of the same genotype (unpaired t-test or Wilcoxon test). ^*^ Significant difference (*p* < 0.05) between genotypes and *Nphs2*^Δipod^ (ANOVA with Dunnetts multiple comparison test or Kruskal-Wallis with Dunn’s multiple comparison test)

In Western blot from plasma samples of uninduced *Nphs2*^Δipod^ mice, FB was detectable at 100 kDa representing the zymogen form and FD at 38–42 kDa [26] under both reducing and non-reducing conditions (Figs. 1a–d, Suppl. Fig. 2). In nephrotic *Nphs2*^Δipod^ mice, FB expression was not appreciably altered whereas it was higher in mice lacking C3 under both uninduced and nephrotic conditions (Fig. 1e). In contrast, plasma FD expression was significantly reduced in nephrotic *Nphs2*^Δipod^ mice (Fig. 1f). Using an antibody against the C-terminus of the α-chain of C3, bands at 145 and 140 kDa under reducing conditions were possibly native C3 or C3 aggregated with certain serum proteins (Fig. 1a, Suppl. Fig. 1). In addition, a band at 115 kDa was detected that most likely represents the intact α-chain of C3. Furthermore, a strong band at 43 kDa was detectable most likely representing fragment 2 of the α´-chain derived from C3c (Suppl. Fig. 1). The appearance of this band most likely reflects spontaneous activation of the alternative complement pathway (so-called tick over). In addition, a band was seen at 45 kDa representing fragment 2 of the α´-chain likely attached with C3f and a band at 38 kDa that most likely represents another degradation product of fragment 2 of the α´-chain. The expression of the C3 band at 146 kDa and that of the α chain of C3 at 115 kDa were increased in nephrotic *Nphs2*^Δipod^ mice, whereas the expression of α´-chain fragment 2 was unaltered. The expression pattern was not appreciably different in *Nphs2*^Δipod^*Cfb^*−/−*^, *Nphs2*^Δipod^*Cfd^*−/−*^ and *Nphs2*^Δipod^*C3^*−/−*^ mice except for the deleted proteins (Figs. 1g–i).

Under non-reducing conditions, bands were in the range of from 125 to 280 kDa and the expression of α´-chain fragments were absent, indicating that these remained attached via disulfide bonds (Fig. 1b, Suppl. Fig. 1). Again, there seemed not to be a difference in the expression pattern between the genotypes.

These results confirmed that our cross-breeding successfully produced targeted mice with deficiencies in C3, FD, or FB in the context of mice with inducible podocin deficiency. Unexpectedly, C3 activation as represented by the expression of fragment 2 of the α´-chain derived from C3c was not absent in FB- or FD-deficient mice both under uninduced and nephrotic conditions.

### The alternative complement pathway is activated in the urine of nephrotic mice irrespective of FB and FD abundance

**Fig. 2:**
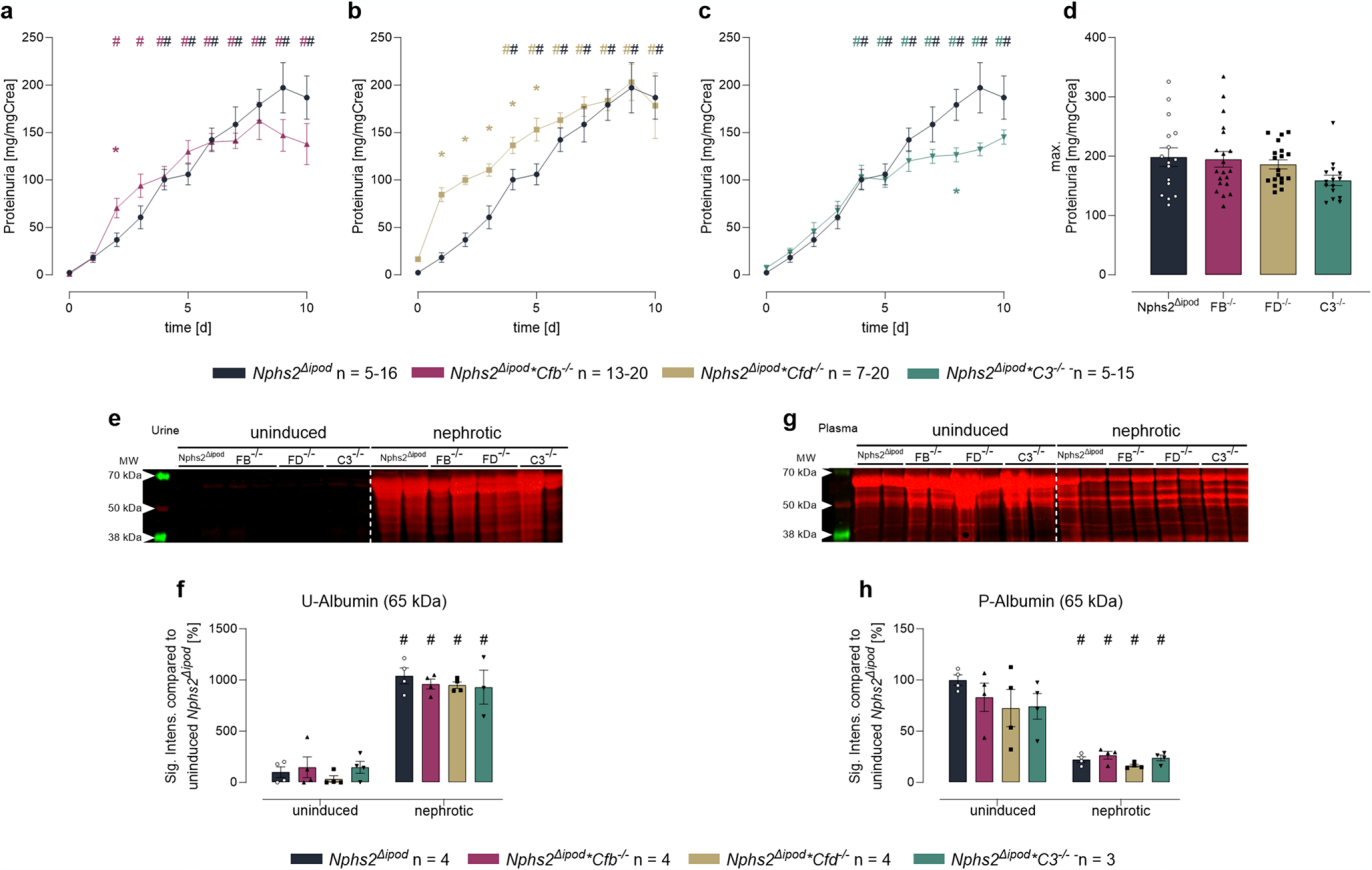
Induction of nephrotic syndrome in *Nphs2*^Δipod^*, Nphs2*^Δipod^*Cfb^-/-^*(FB*^-/-^*), Nphs2*^Δipod^*Cfd^-/-^ (FD^-/-^) and* Nphs2*^Δipod^*C3^-/-^(C3^-/-^) mice. **a–c** Course of proteinuria after end of induction treatment at day 0. **d** maximal proteinuria normalized for urinary creatinine concentration after 8 days. **e** Western blot of urine samples after total protein staining. Note the albuminuria at 65 kDa after induction of nephrotic syndrome. **f** Densitometry of albumin abundance before and after induction of nephrotic syndrome. **g** Western blot of plasma samples after total protein staining. **h** Densitometry of albumin abundance before and after induction of nephrotic syndrome. Trajectories of *Nphs2*^Δipod^ mice are pooled and results with the respective knockout line were plotted separately for clarity. ^#^ Significant difference (*p* < 0.05) between uninduced and nephrotic mice of the same genotype (unpaired t-test or Wilcoxon test). ^*^ Significant difference (*p* < 0.05) between genotypes and *Nphs2*^Δipod^ (ANOVA with Dunnetts multiple comparison test or Kruskal-Wallis with Dunn’s multiple comparison test)

To study whether C3, FB and FD are aberrantly filtered, activated and excreted in the urine, we induced experimental nephrotic syndrome in *Nphs2*^Δipod^*Cfb^*−/−*^, *Nphs2*^Δipod^*Cfd^*−/−*^ and *Nphs2*^Δipod^*C3^*−/−*^ mice and their *Nphs2*^Δipod^ littermates. As shown in Figs. 2a–e, all genotypes developed nephrotic proteinuria and albuminuria that approached a similar level. The onset of proteinuria was accelerated in *Nphs2*^Δipod^*Cfd^*−/−*^ mice (Fig. 2b). In all genotypes, this was accompanied by a similar reduction in plasma albumin abundance (Fig. 2g).

**Fig. 3:**
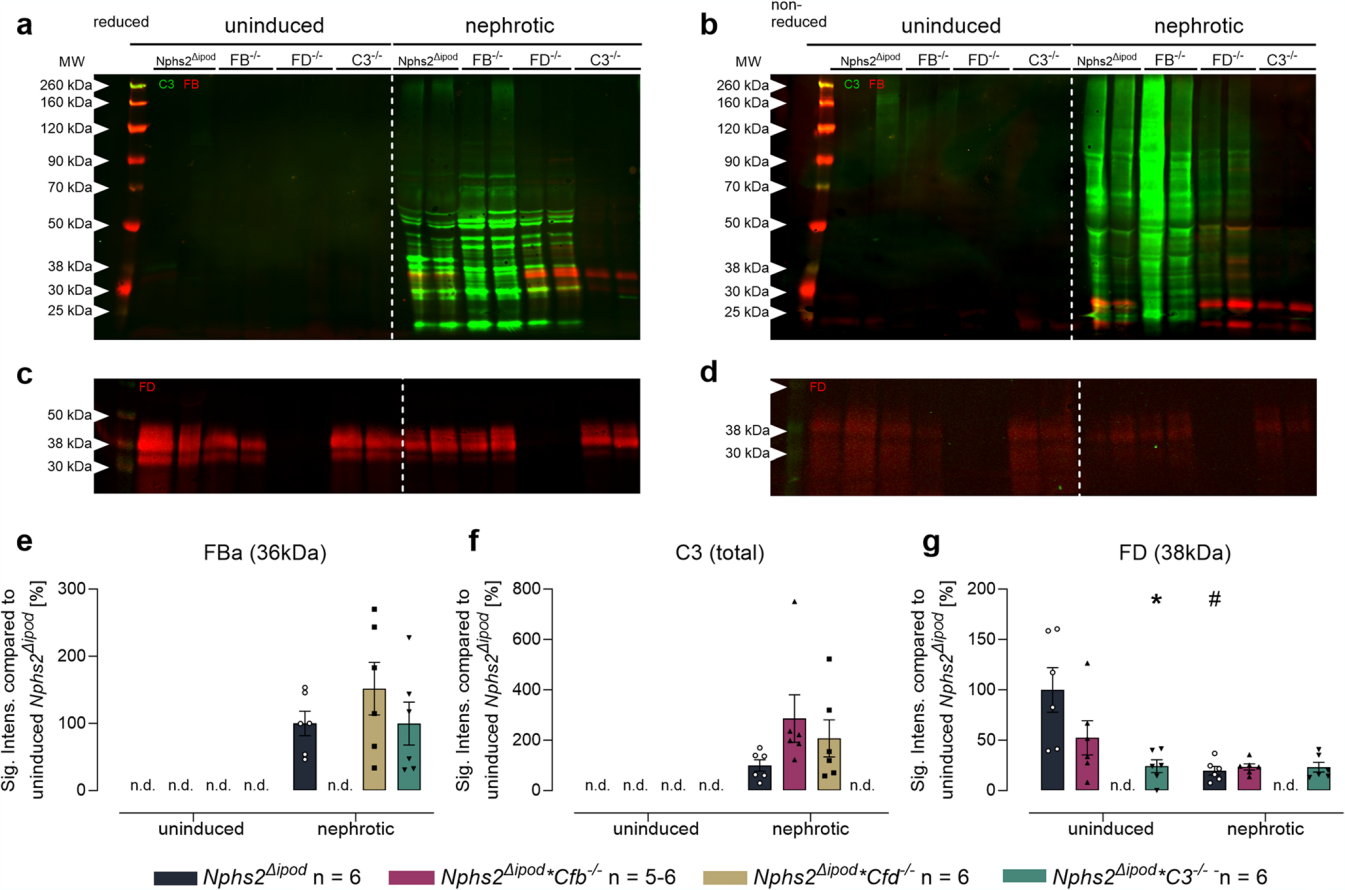
Expression of FB, FD and C3 in the urine of *Nphs2*^Δipod^, *Nphs2*^Δipod^*Cfb^-/-^*(FB*^-/-^*)*, *Nphs2*^Δipod^*Cfd^-/-^ (FD^-/-^) and *Nphs2*^Δipod^*C3^-/-^(C3^-/-^) mice before and after induction of experimental nephrotic syndrome. **a, b** Western blot for expression of C3 (green) and FB (red) under reducing **a** or non-reducing conditions **b**. **c, d** Western blot for expression of FD (red) under reducing **c** or non-reducing conditions **d**. Note that the signal is weaker under non-reducing conditions, suggesting reduced recognition of FD by the antibody. **e–i** Densitometry of the obtained bands under reducing conditions. Note the specificity of the antibodies used as they lack any signal in the respective ko strain. ^#^ Significant difference (*p* < 0.05) between uninduced and nephrotic mice of the same genotype (unpaired t-test or Wilcoxon test). ^*^ Significant difference (*p* < 0.05) between genotypes and *Nphs2*^Δipod^ (ANOVA with Dunnetts multiple comparison test or Kruskal-Wallis with Dunn’s multiple comparison test)

In uninduced *Nphs2*^*Δipod*^ mice there was no excretion of FB and C3 in the urine in contrast to FD which was detectable owing to its low molecular weight [17] (Figs. 3a–c, Suppl. Fig. 2). In nephrotic *Nphs2*^*Δipod*^ mice, C3 appeared in the urine, however, the band pattern was strongly different to the results obtained from plasma. Under reducing conditions, there were multiple bands of C3 in the low molecular range which were also present under non-reducing conditions, indicating the proteolysis at multiple sites and the appearance of fragments that were no longer attached via the disulfide bonds (Figs. 3a, b). Under reducing and non-reducing conditions, FB was detected predominantly as Ba fragment at 40 kDa and additional smaller fragments (Figs. 3a, b). The appearance of FD was similar in nephrotic *Nphs2*^*Δipod*^ mice compared to the induced state (Figs. 3c, d). Unexpectedly, C3 degradation products seemed to be stronger expressed in nephrotic *Nphs2*^Δipod^*Cfb^−/−^ and *Nphs2*^Δipod^*Cfd^−/−^ mice (Figs. 3a, b, f). Overall, these Western blot results suggest that C3 and FB as major components of the alternative complement pathway are aberrantly filtered into the urine of nephrotic mice and undergo extensive proteolytic processing and degradation. Regarding degradation of C3, FB and FD seemed to be dispensable.

**Fig. 4:**
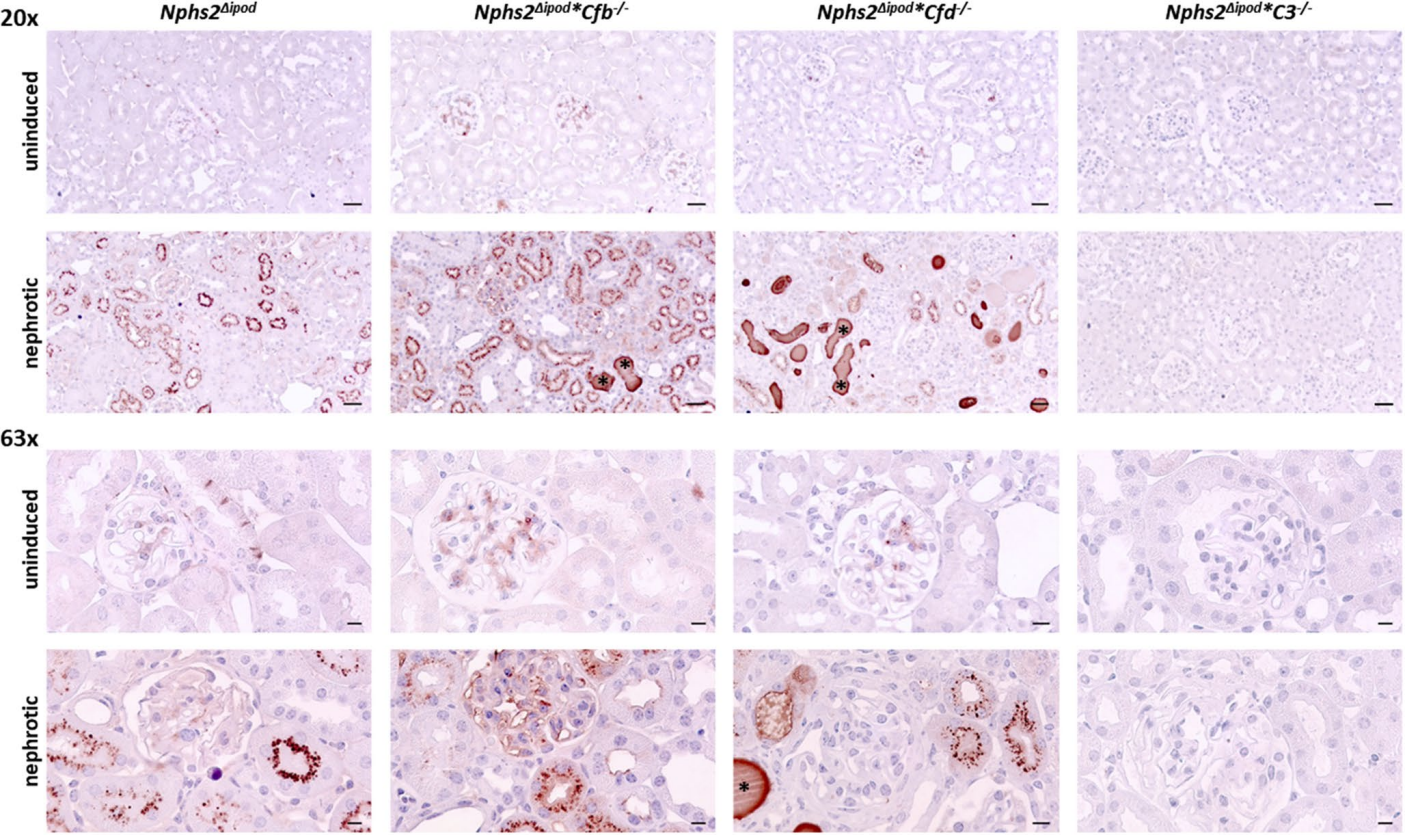
Tissue expression of C3 in *Nphs2*^Δipod^, *Nphs2*^Δipod^*Cfb^-/-^, *Nphs2*^Δipod^*Cfd^-/-^ and *Nphs2*^Δipod^*C3^-/-^ mice before and after induction of nephrotic syndrome. Representative staining of kidney sections stained for C3 at 20- (upper panel, scale 20µm) and 63-fold (lower panel, scale 5µm) magnification. The antibody was the same as used for Western blot. No signal is obtained in *Nphs2*^Δipod^*C3^-/-^ mice. * marks the C3-positive tubular protein casts

Tissue expression of C3 was analyzed using immunohistochemistry. As shown in Fig. 4, the staining was negative in uninduced *Nphs2*^*Δipod*^ mice except for minimal trapping of C3 in glomeruli most likely due to incomplete perfusion. In contrast, nephrotic *Nphs2*^*Δipod*^ mice showed strong signals of vesicular appearance within the tubular cells, most likely due to avid uptake C3 fragments of low molecular weight by the proximal tubule. This pattern was accentuated in nephrotic FB and FD-deficient mice and in addition there were strongly stained C3-positive tubular protein casts. Noteworthy, in nephrotic mice of all genotypes C3 staining did not involve the glomeruli indicating the non-inflammatory nature of the experimental nephrotic model.

## Nephrotic mice deficient in C3, FB or FD experience similar ENaC activation and sodium retention

**Fig. 5:**
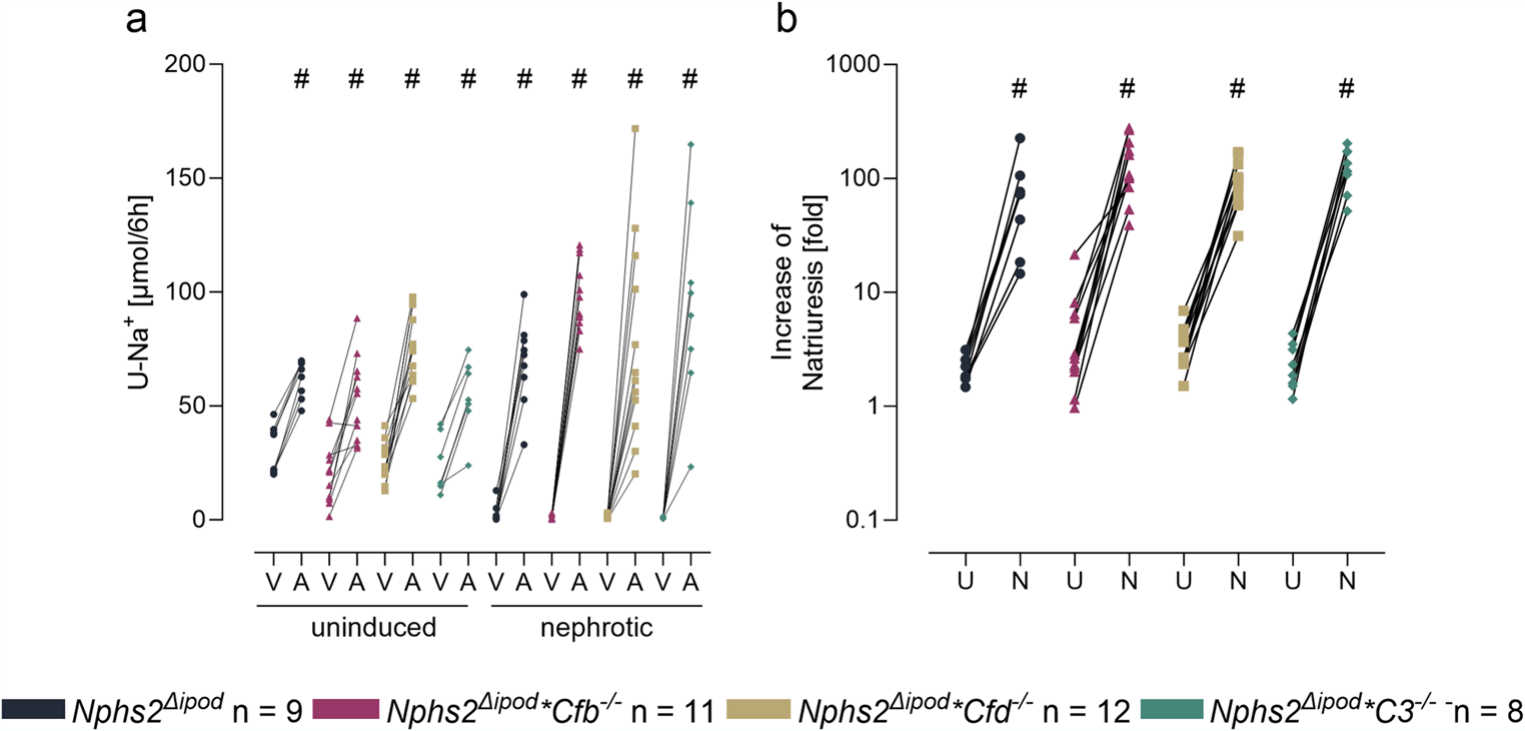
Amiloride-sensitive natriuresis in *Nphs2*^Δipod^, *Nphs2*^Δipod^*Cfb^-/-^, *Nphs2*^Δipod^*Cfd^-/-^ and *Nphs2*^Δipod^*C3^-/-^ mice before and after induction of nephrotic syndrome. **a** Natriuretic response to the acute administration of the ENaC inhibitor amiloride (A, 10 µg/g) or vehicle injection (V, injectable water, 5µl/g). **b** Fold-increase of the natriuretic response after amiloride administration before (uninduced, U) and after (nephrotic, N) induction of nephrotic syndrome. ^#^ Significant difference (*p* < 0.05) between uninduced and nephrotic mice of the same genotype (unpaired t-test or Wilcoxon test). ^*^ Significant difference (*p* < 0.05) between genotypes and *Nphs2*^Δipod^ (ANOVA with Dunnetts multiple comparison test or Kruskal-Wallis with Dunn’s multiple comparison test)

We then focused on studying the impact of the excretion and activation of C3, FB and FD on ENaC-mediated sodium retention in experimental nephrotic syndrome. To investigate ENaC activity, we determined the response to a single dose of the ENaC blocker amiloride before and after induction of nephrotic syndrome. As shown in Fig. 5a, the natriuretic response was similar in all genotypes in the healthy uninduced state. After induction of nephrotic syndrome, natriuretic response to amiloride increased significantly in all genotypes reaching similar values. ENaC activation in nephrotic mice was most evident when the ratio of natriuresis between vehicle and amiloride was calculated showing a significant increase in all genotypes compared to the values obtained under control conditions (Fig. 5b).

**Fig. 6:**
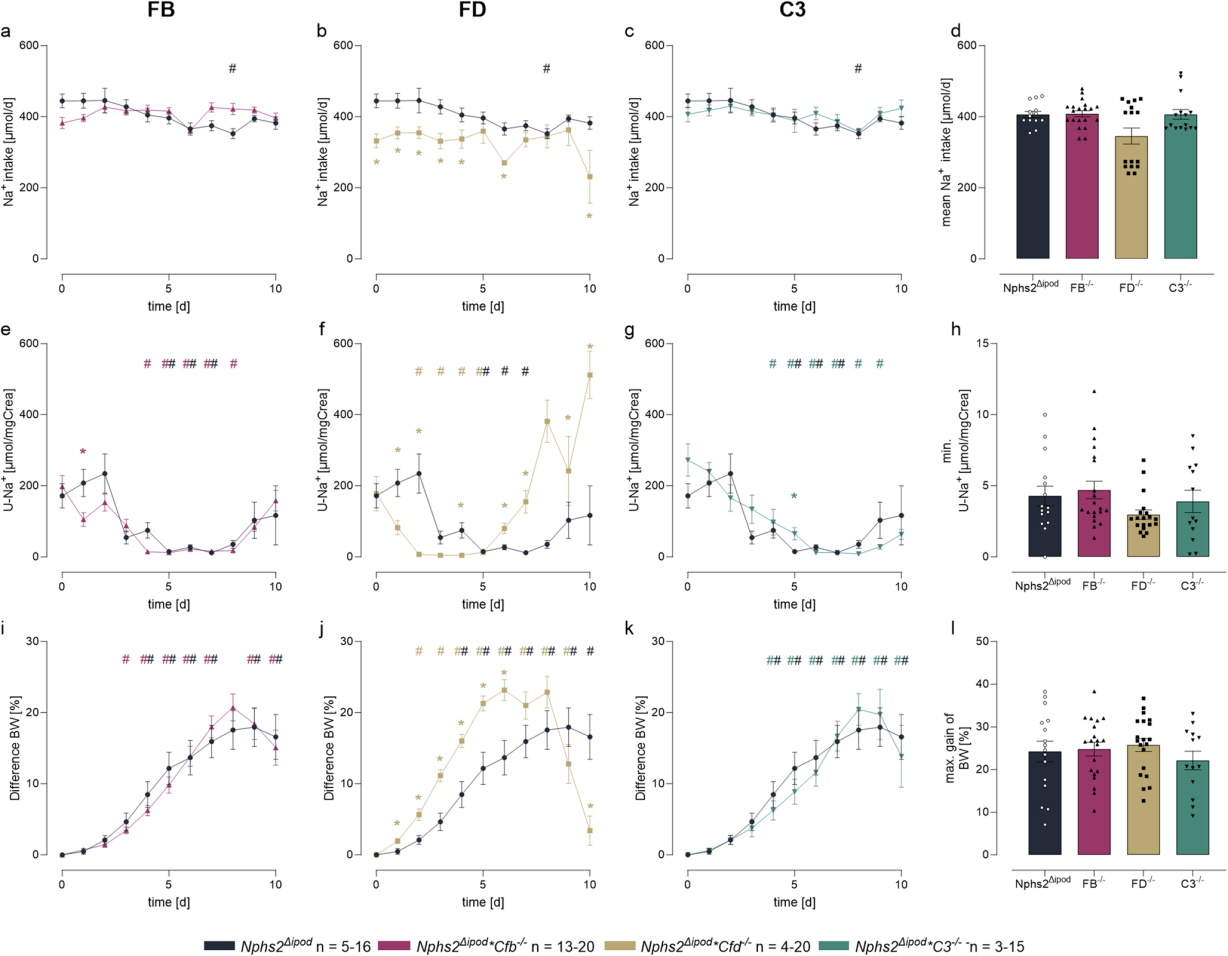
Sodium retention in *Nphs2*^Δipod^, *Nphs2*^Δipod^*Cfb^-/-^*(FB*^-/-^*), Nphs2*^Δipod^*Cfd^-/-^ *(FD*^-/-^) and * Nphs2*^Δipod^*C3^-/-^(C3^-/-^) mice after induction of nephrotic syndrome. Course of sodium intake **a–c**, urinary sodium excretion in spot urine samples **e–g** and body weight **i–k** after induction of nephrotic syndrome. **d** arithmetic mean of sodium intake. **h**, **l** minimal urinary sodium excretion **h** and maximal body weight gain **l**, both reflecting maximal ENaC activation. ^#^ Significant difference (*p* < 0.05) between uninduced and nephrotic mice of the same genotype (unpaired t-test or Wilcoxon test) ^*^ Significant difference (*p* < 0.05) between genotypes and *Nphs2*^Δipod^ (ANOVA with Dunnetts multiple comparison test or Kruskal-Wallis with Dunn’s multiple comparison test)

During the course of experimental nephrotic syndrome food and fluid intake was constant (Suppl. Fig. 3) as was the calculated sodium intake (Figs. 6a–d). However, daily urinary sodium concentration dropped in all genotypes to values < 20 mM or < 15 µmol/mg creatinine (Figs. 6e–h). The positive sodium balance was also evident from studies of nephrotic mice in metabolic cages (Table 1). Subsequently, nephrotic mice of all genotypes gained body weight and developed ascites indicating sodium retention (Figs. 6i–k). The maximal body weight gain was 24 ± 2% in *Nphs2*^*Δipod*^, 25 ± 2% in *Nphs2*^Δipod^*Cfb^*−/−*^, 22 ± 3% in *Nphs2*^Δipod^*Cfd^*−/−*^ and 26 ± 2% in *Nphs2*^Δipod^*C3^*−/−*^ mice, respectively, which was not significantly different (*p* = 0.398, Fig. 6l). Thereafter, in all genotypes urinary sodium excretion started to increase spontaneously, paralleled by reversal of body weight gain (Figs. 6e–k). This phenomenon is a characteristic feature of experimental NS in rodents which remains poorly understood [27]. In *Nphs2*^Δipod^*Cfd^*−/−*^ mice, this reversal was accelerated, leading to almost complete normalization of body weight at day 10 (Fig. 6j).

**Table 1:** Sodium balance in *Nphs2*^Δipod^*, Nphs2*^Δipod^*Cfb^-/-^, *Nphs2*^Δipod^*Cfd^-/-^ and *Nphs2*^Δipod^*C3^-/-^ mice before and after induction of experimental nephrotic syndrome

	Healthy				Nephrotic			
	*Nphs2* ^Δipod^	*Nphs2* ^Δipod^ *Cfb ^-/-^	*Nphs2* ^Δipod^ *Cfd ^-/-^	*Nphs2* ^Δipod^ *C3 ^-/-^	*Nphs2* ^Δipod^	*Nphs2* ^Δipod^ *Cfb ^-/-^	*Nphs2* ^Δipod^ *Cfd ^-/-^	*Nphs2* ^Δipod^ *C3 ^-/-^
Na^+^ intake [µmol/24 h]	286 ± 13	285 ± 14	313 ± 14	287 ± 9	322 ± 14^#^	349 ± 12^#^	332 ± 13	335 ± 7^#^
urinary Na^+^ excr. [µmol/24 h]	182 ± 16	117 ± 8^*^	130 ± 12	130 ± 7	11 ± 3^#^	11 ± 1^#^	26 ± 5^*#^	11 ± 1^#^
fecal Na^+^ excr. [µmol/24 h]	8 ± 2	19 ± 2^*^	25 ± 3^*^	18 ± 3	5 ± 1	21 ± 2^*^	9 ± 1^#^	24 ± 1^*^
Na^+^ balance [µmol/24 h]	95 ± 13	149 ± 15^*^	158 ± 7^*^	139 ± 9	304 ± 12^#^	315 ± 14^#^	295 ± 12^#^	301 ± 6^#^

Values for nephrotic mice were taken on day 8 of nephrotic syndrome

Arithmetic means ± SEM (n= 8-11 each)

^#^ Significant difference (*p* < 0.05) between uninduced and nephrotic mice of the same genotype, (Wilcoxon test)

^*^ significant difference (*p* < 0.05) between genotypes and *Nphs2*^Δipod^ (Kruskal-Wallis with Dunn’s multiple comparison test)

Table 2 depicts the plasma concentrations of electrolytes, hematocrit and plasma urea concentration. In the uninduced state, there was no difference between the genotypes except for a slight acidosis in *Nphs2*^Δipod^*Cfb^*−/−*^ mice. After induction of nephrotic syndrome, *Nphs2*^*Δipod*^ mice experienced a drop in plasma sodium concentration and hematocrit, increase in plasma potassium and standard bicarbonate concentration. Renal function was mildly reduced as evidenced from an increase in plasma urea concentration. These changes were similar in nephrotic *Nphs2*^Δipod^*Cfb^*−/−*^, *Nphs2*^Δipod^*Cfd^*−/−*^ and *Nphs2*^Δipod^*C3^*−/−*^ mice.

**Table 2:** Plasma parameters obtained from *Nphs2*^Δipod^*, Nphs2*^Δipod^*Cfb^-/-^, *Nphs2*^Δipod^*Cfd^-/-^ and * Nphs2*^Δipod^*C3^-/-^ mice before and after induction of experimental nephrotic syndrome

	Healthy				Nephrotic			
	*Nphs2*^Δipod^	*Nphs2*^Δipod^*Cfb^-/-^	*Nphs2*^Δipod^*Cfd^-/-^	*Nphs2*^Δipod^*C3^-/-^	*Nphs2*^Δipod^	*Nphs2*^Δipod^*Cfb^-/-^	*Nphs2*^Δipod^*Cfd^-/-^	*Nphs2*^Δipod^*C3^-/-^
pH	7.26 ± 0.01	7.21 ± 0.01*	7.24 ± 0.01	7.25 ± 0.01	7.30 ± 0.01^#^	7.31 ± 0.01^#^	7.35 ± 0.004*^#^	7.28 ± 0.01^#^
Na^+^ [mmol/l]	150 ± 1	151 ± 1	149 ± 1	150 ± 1	147 ± 1^#^	143 ± 3	142 ± 2^#^	146 ± 1^#^
K^+^ [mmol/l]	4.5 ± 0,1	4.5 ± 0,1	4.4 ± 0,1	5.1 ± 0,2	5.2 ± 0.2^#^	5.3 ± 0.1^#^	4.9 ± 0.1^#^	5.3 ± 0.1
Hct [%]	47 ± 1	50 ± 1*	47 ± 1	50 ± 1	42 ± 1^#^	42 ± 1^#^	43 ± 2	46 ± 1^#^
HCO_3_^-^std [mmol/l]	22 ± 0.5	19 ± 0.3*	21 ± 0.4	22 ± 0.5	24 ± 0.5^#^	27 ± 1.2*^#^	28 ± 0.6*^#^	24 ± 0.5^#^
urea [mg/dl]	34 ± 2	34 ± 4	37 ± 1	38 ± 2	63 ± 3^#^	63 ± 5^#^	81 ± 1*^#^	60 ± 6^#^

Arithmetic means ± SEM (n=8-11 each)

^#^ Significant difference (*p* < 0.05) between uninduced and nephrotic mice of the same genotype (unpaired t-test or Wilcoxon test)

^*^ Significant difference (*p* < 0.05) between genotypes and *Nphs2*^Δipod^ (ANOVA with Dunnetts multiple comparison test or Kruskal-Wallis with Dunn’s multiple comparison test)

Abbreviations: *std* standard, *Hct* hematocrit

### Apical targeting and proteolytic processing of γ-ENaC is not altered in nephrotic mice deficient for C3, FB or FD

**Fig. 7:**
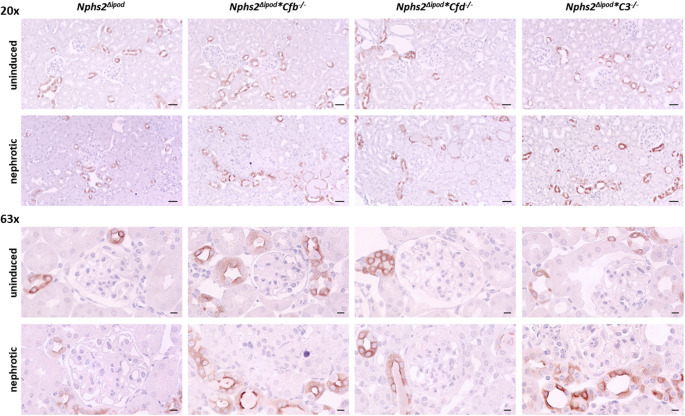
Tissue expression of γ-ENaC in *Nphs2*^Δipod^, *Nphs2*^Δipod^*Cfb^-/-^, *Nphs2*^Δipod^*Cfd^-/-^ and *Nphs2*^Δipod^*C3^-/-^ mice before and after induction of nephrotic syndrome. Representative staining of kidney sections stained for γ-ENaC at 20- (upper panel, scale 20µm) and 63-fold (lower panel, scale 5µm) magnification. The antibody was the same as used for Western blot

ENaC activity is strongly determined by apical targeting to the luminal plasma membrane and proteolytic processing. To study the tissue expression of γ-ENaC in the kidney we performed immunohistochemistry. In uninduced *Nphs2*^*Δipod*^ mice, immunohistochemical γ-ENaC staining was characterized by a predominantly cytosolic pattern (Fig. 7 [28]). After induction of nephrotic syndrome, the expression of γ-ENaC shifted to the apical plasma membrane, as previously shown and known as apical targeting [29, 30]. This expression pattern was unaltered in uninduced and nephrotic *Nphs2*^Δipod^*Cfb^*−/−*^, *Nphs2*^Δipod^*Cfd^*−/−*^ and *Nphs2*^Δipod^*C3^*−/−*^ mice.

**Fig. 8:**
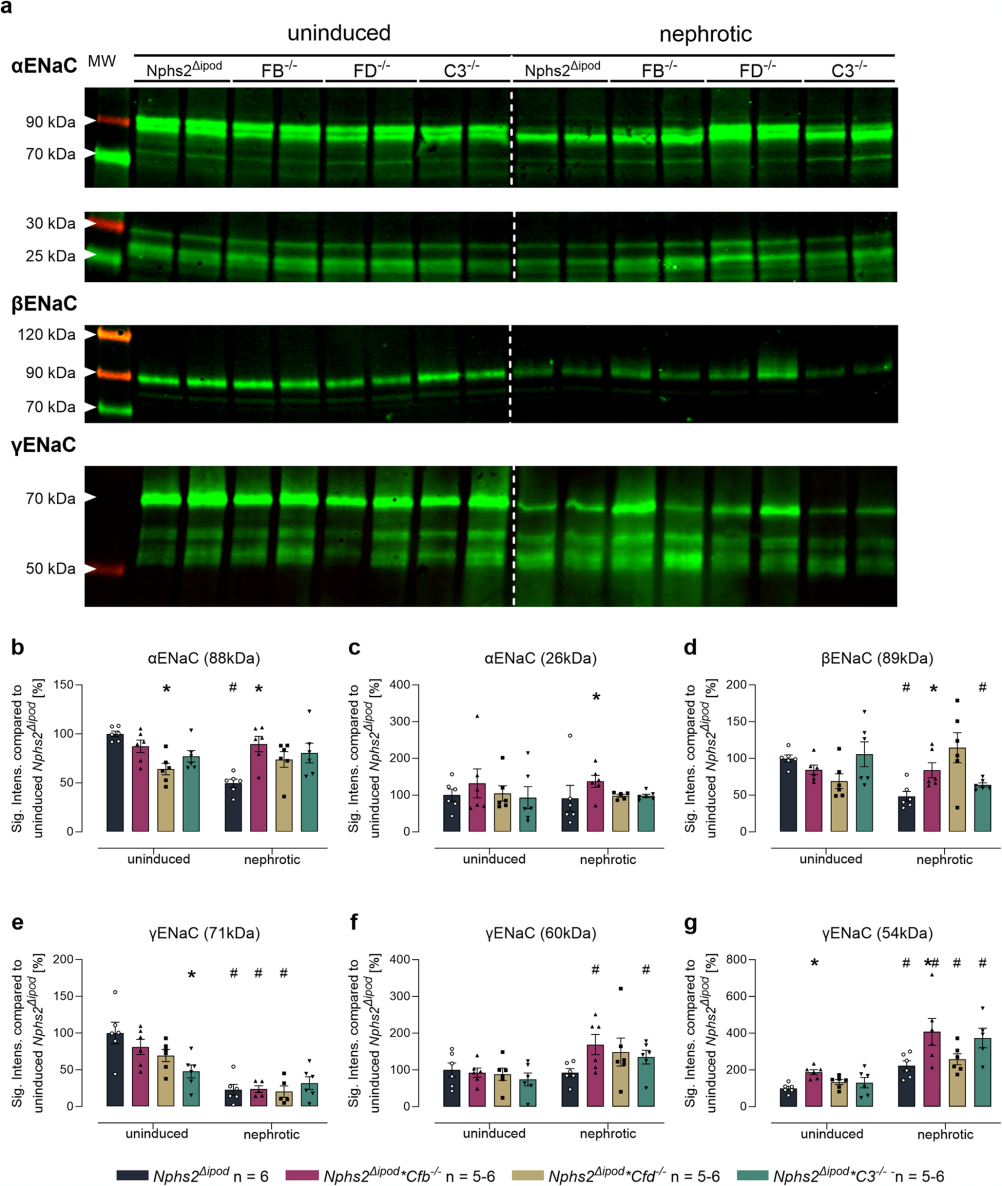
Expression of ENaC subunits and proteolytic processing in kidney lysates from *Nphs2*^Δipod^*, Nphs2*^Δipod^*Cfb^-/-^*(FB*^-/-^*), Nphs2*^Δipod^*Cfd^-/-^ (FD^-/-^) and* Nphs2*^Δipod^*C3^-/-^(C3^-/-^) mice before and after induction of nephrotic syndrome. **a** Representative Western blots showing the expression of α-, β- and γ-ENaC in a plasma membrane preparation of kidney cortex lysates before (healthy) and after induction (nephrotic) of nephrotic syndrome. Note that the samples were deglycosylated before analyzing expression of γ-ENaC and its cleavage products [25]. The white line is only for optical discrimination, it is one blot each, no vertical cutting. **b–g** Densitometry of the obtained bands normalized for total protein content of each lane. ^#^ Significant difference (*p* < 0.05) between uninduced and nephrotic mice of the same genotype (unpaired t-test or Wilcoxon test) ^*^ Significant difference (*p* < 0.05) between genotypes and *Nphs2*^Δipod^ (ANOVA with Dunnetts multiple comparison test or Kruskal-Wallis with Dunn’s multiple comparison test)

We then studied the expression and proteolytic processing of ENaC subunits using Western blot. In kidney cortex lysates from uninduced *Nphs2*^*Δipod*^ mice, Western blot analyses identified two bands for α-ENaC at 88 and 26 kDa corresponding to full-length and a cleavage product after distal cleavage (designated from the N-terminus; Fig. 8a, Suppl. Fig 2). For β-ENaC, there was only a single band at 89 kDa corresponding to the full-length subunit which is not proteolytically processed (Fig. 8a). For γ-ENaC there were three bands in deglycosylated samples at 71, 60 and 54 kDa (Fig. 8a) corresponding to full-length, proximally and distally cleaved fragments, respectively [24, 25]. In uninduced mice of all genotypes, there were no significant differences in the expression of any ENaC subunit except for a lower expression of full-length γ-ENaC in *Nphs2*^Δipod^*Cfd^*−/−*^ mice. After induction of nephrotic syndrome, the expression of full-length α-, β- and γ-ENaC decreased in *Nphs2*^*Δipod*^ mice, however, the expression of proximally (60 kDa) and distally cleaved (54 kDa) γ-ENaC was increased (Fig. 8f, g). In nephrotic *Nphs2*^Δipod^*Cfb^*−/−*^, *Nphs2*^Δipod^*Cfd^*−/−*^ and *Nphs2*^Δipod^*C3^*−/−*^ mice, there were large variations in the expression of ENaC subunits in both directions, however, the increased expression of proximally and distally cleaved γ-ENaC was consistent. Overall, the Western blot results confirm that ENaC was proteolytically processed in the absence of the serine proteases FB and FD as well as C3.

## Discussion

The present study confirms that the complement factors C3 and FB of the alternative complement pathway are excreted in the urine after induction of experimental nephrotic syndrome. Moreover, urine contains fragments of these factors, including α’ chain fragment 2 derived from C3c and Ba, indicating proteolytic processing and activation. For C3, we saw avid tubular reabsorption of these fragments. These findings indicate the intratubular activation of the alternative complement pathway in experimental nephrotic syndrome. From the biology of the complement system, the absence of FB and FD should, in theory, result in a mitigated activation of C3 through alternative pathway but the classical pathway and the lectin pathway still remained intact. However, our Western blot data from mice lacking FB and FD indicate that C3 was also activated in the absence of these serine proteases, suggesting the action of other proteases or other pathways. Besides activation of C3 with appearance of characteristic fragments such as the fragment 2 of the alpha´ chain derived from C3c (Suppl. Fig. 1), we found numerous other unknown degradation products of C3 in the urine, indicating complex and unconventional proteolytic events. A recent study found that plasmin derived from plasminogen after activation by uPA was able to degrade complement factors C3 and also C5 when incubated in purified form in vitro [19]. The band pattern was similar to our results obtained from urine samples, demonstrating multiple degradation products of C3. Using the same mouse model, the authors reported that intratubular complement activation can be reduced by inhibition of uPA. It must be underscored that plasmin is quantitatively the most abundant serine protease excreted in urine samples from nephrotic mice, which reflects its high plasma concentration in comparison to other serine proteases from the coagulation and complement system [11]. Therefore, it is conceivable that plasmin might be one of the drivers of C3 activation in nephrotic syndrome, although other proteases capable of activating C3 might act in concert and have been reported to be excreted in nephrotic urine such as thrombin, coagulation factor X [31] or plasma kallikrein [32]. In conclusion, the findings are consistent with the notion that nephrotic syndrome leads to a burst of urinary protease activity, previously termed proteasuria [3, 11].

In nephrotic mice, pharmacological inhibition of urinary serine protease activity by the use of aprotinin prevented proteolytic activation of ENaC and sodium retention, providing evidence that proteasuria is not just a descriptive term but a mediator of edema formation in NS [6, 7, 9]. Since then, the identification of the relevant serine proteases has been an ongoing quest [33] and mice lacking various aprotinin-sensitive serine proteases from the coagulation cascade including uPA or plasminogen were not protected from edema formation in experimental NS [8, 9, 12, 13]. Given the urinary excretion of FB and FD in experimental NS and the emerging role of oral complement inhibitors such as iptacopan for the inhibition of FB or danicopan for the inhibition of FD, it was imperative to test these serine proteases with regard to their impact on ENaC-mediated sodium retention in experimental NS. Unlike in humans [17], we observed urinary excretion of the serine protease FD in uninduced *Nphs2*^Δipod^ mice, highlighting a key difference between the mouse and human complement systems. The molecular weight of human FD is 25 kDa, whereas in mice it is 38–42 kDa due to glycosylation. Because of its low molecular weight, FD can be filtered through the glomeruli. Interestingly, we observed that the urine of normal mice contains a large amount of FD. Whether these FD participate in downstream complement-mediated immune responses in the urinary tract warrants further investigation.

The current study clearly indicates that a lack of both FD and FB does not confer protection against sodium retention and proteolytic processing of γ-ENaC. Furthermore, the lack of C3 as the major hub of the complement system including both the alternative and classical pathways was also not protective. These results suggest that intratubular activation of the proximal complement system is dispensable for sodium retention in NS. Translating these findings to treatment of nephrotic patients predicts that oral complement inhibitors are not expected to have an effect on sodium retention and edema formation, which are common findings in proteinuric glomerulopathies such as C3 glomerulopathy and IgA nephropathy for which iptacopan was recently approved. In theory, our data cannot exclude a potential impact of the terminal phase of the complement system represented by C5 or the membrane attack complex C5b-9. However, the latter does not exert a protease activity and is not expected to activate ENaC proteolytically.

In conclusion, we demonstrate that components of the alternative complement pathway are detectable and activated in nephrotic syndrome. Mice with deletion of C3, FB or FD were not protected from proteolytic ENaC activation and sodium retention in NS.

## Supplementary information

Below is the link to the electronic supplementary material.

Supplementary file 1 (DOCX 1.70 MB)

## Data Availability

Data will be shared upon reasonable request.
