# Supplementary material for: Activation of the alternative complement pathway and its relevance for sodium retention in experimental nephrotic syndrome
Source: Pflugers Arch. 2026 Jan 3;478(1):16. doi: 10.1007/s00424-025-03136-x (PMC12764515; doi:10.1007/s00424-025-03136-x)
Supplement: Supplementary file 1 — (DOCX 1.70 MB) [file 424_2025_3136_MOESM1_ESM.docx]

**Supplement:**

**Supplemental Table 1. Used primers.**

| **gene** | **sense/forward**  **5’→3’ orientation** | **antisense/reverse**  **5’→3’ orientation** | **amplicon** | **reference** |
| --- | --- | --- | --- | --- |
| Nphs2^tm3.1Antc^ | CCA GCA TCC CAT TAG ATA GAT GAG G | GCA TCC AAA TGA TCA GAG TTC CCA GG | 236 / 286 bp (wt / floxed allele) | [1] |
| Tg(Nphs1-rtTA*3G)^8Jhm^ | GAA GCA GCA GAA TGA GTT CAC ACT GGG TCC | ACT TTG CTC TTG TCC AGT CTA GAC ATG GTG | 400 bp | [2] |
| Tg(tetO-cre) ^1Jaw^ | GCA TAA CCA GTG AAA CAG CAT TGC TG | GGA CAT GTT CAG GGA TCG CCA GGC G | 280 bp | [3] |
| *C3-wt* | gat ccc cag agc taa tg | agg gac cag ccc agg ttc ag | 373 bp | [4] |
| *C3-ko* | tcg tcc tgc agt tca ttc ag |  | 504 bp |  |
| *CFB-wt* | ccg aag cat tcc tat cct cc | gta gtc ttg tct gct ttc tcc | 751 bp | [5] |
| *CFB-ko* | cga atg ggt gac cgc ttc c |  | 600 bp |  |
| *CFD-wt* | GCG ATG GTA TGA TGT GCA G | GGT TGC TCT CTG CAC ACA T | 464 bp | [6] |
| *CFD-ko* | GGC CGA TCC CAT ATT GGC |  | 320 bp |  |

**Supplemental Table 2: Primary antibodies**

| **antibody** | **host** | **source** | **reference** |
| --- | --- | --- | --- |
| Anti-α-ENaC | rabbit | Pineda antibody service | [7] |
| Anti-β-ENaC | rabbit | Pineda antibody service | [7] |
| Anti-γ-ENaC | rabbit | Stressmarq SPC-405 | [7] |
| Anti-C3 | rabbit | Abcam EPR19394 | [8] |
| Anti-FB | goat | Complement Tech | [9] |
| Anti-FD | sheep | R&D Systems | [10] |

**Supplemental Figure 1: Structure of C3 and its degradation products according to [11]**

The used antibody binds to the C-terminus of C3 detecting α-chain and its degradation products.

The disulfide bonds lead to different band sizes in reducing or non-reducing conditions.


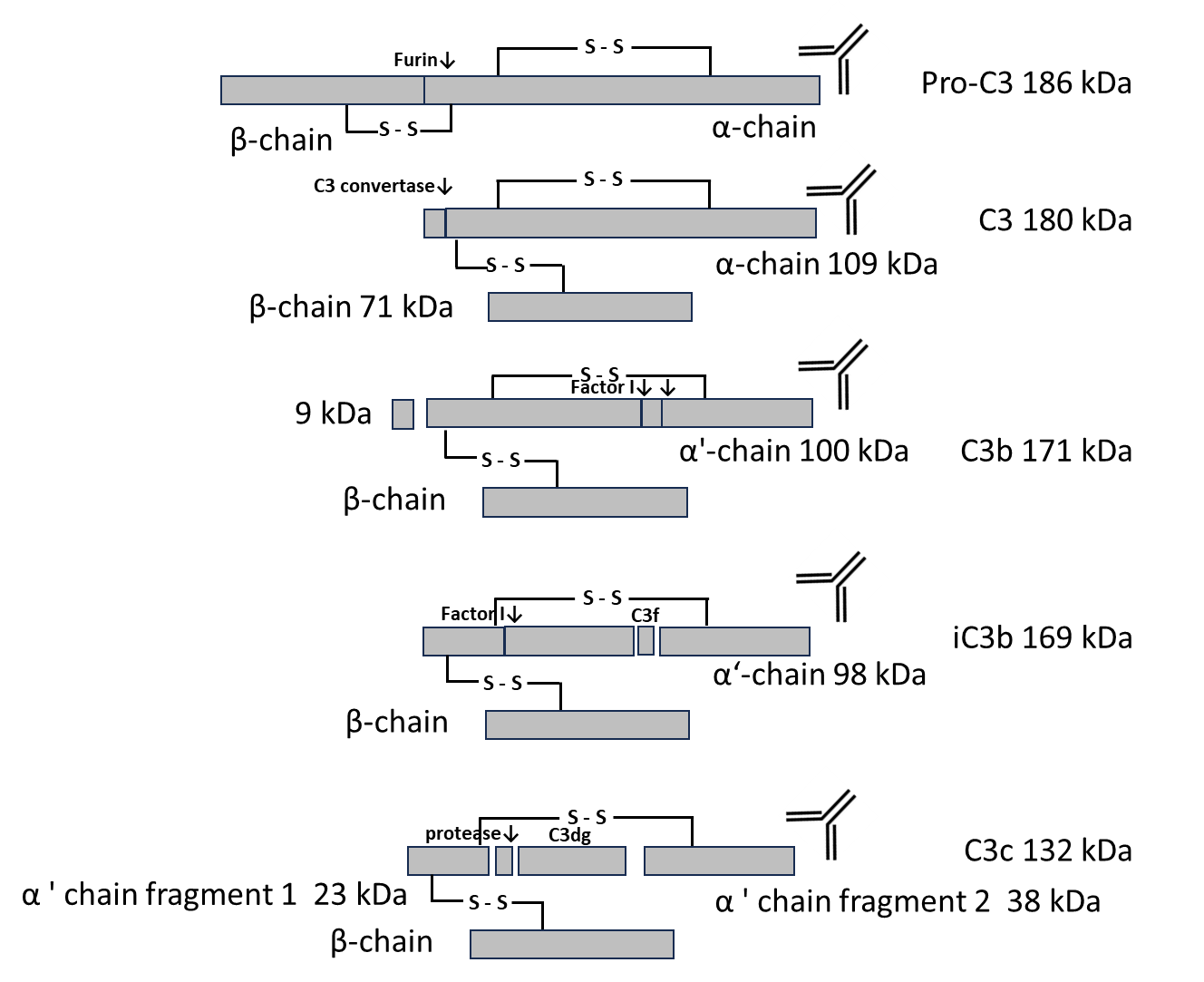


**Supplemental Figure 2: Total protein staining of the western blots shown in Fig. 1, 2 and 8**

**a,b** Total protein staining from the western blot for expression of C3 and FB in the plasma under reducing (**a**) or non-reducing conditions (b)

**c,d** Total protein staining from the western blot for expression of FD in the plasma under reducing (**c**) or non-reducing conditions (d)

**e,f** Total protein staining from the western blot for expression of C3 and FB in the urine under reducing (e) or non-reducing conditions (f)

**g,h** Total protein staining from the western blot for expression of FD in the urine under reducing (g) or non-reducing conditions (h).

**i-k** Total protein staining from the western blot of the expression of α- (i), β- (j) and γ-(k) ENaC in a plasma membrane preparation of kidney cortex lysates before and after induction of nephrotic syndrome.

Note in a-g, i and k there are 17 lanes, the 17^th^ lane (marked as X) was used as loading control and not used for quantification


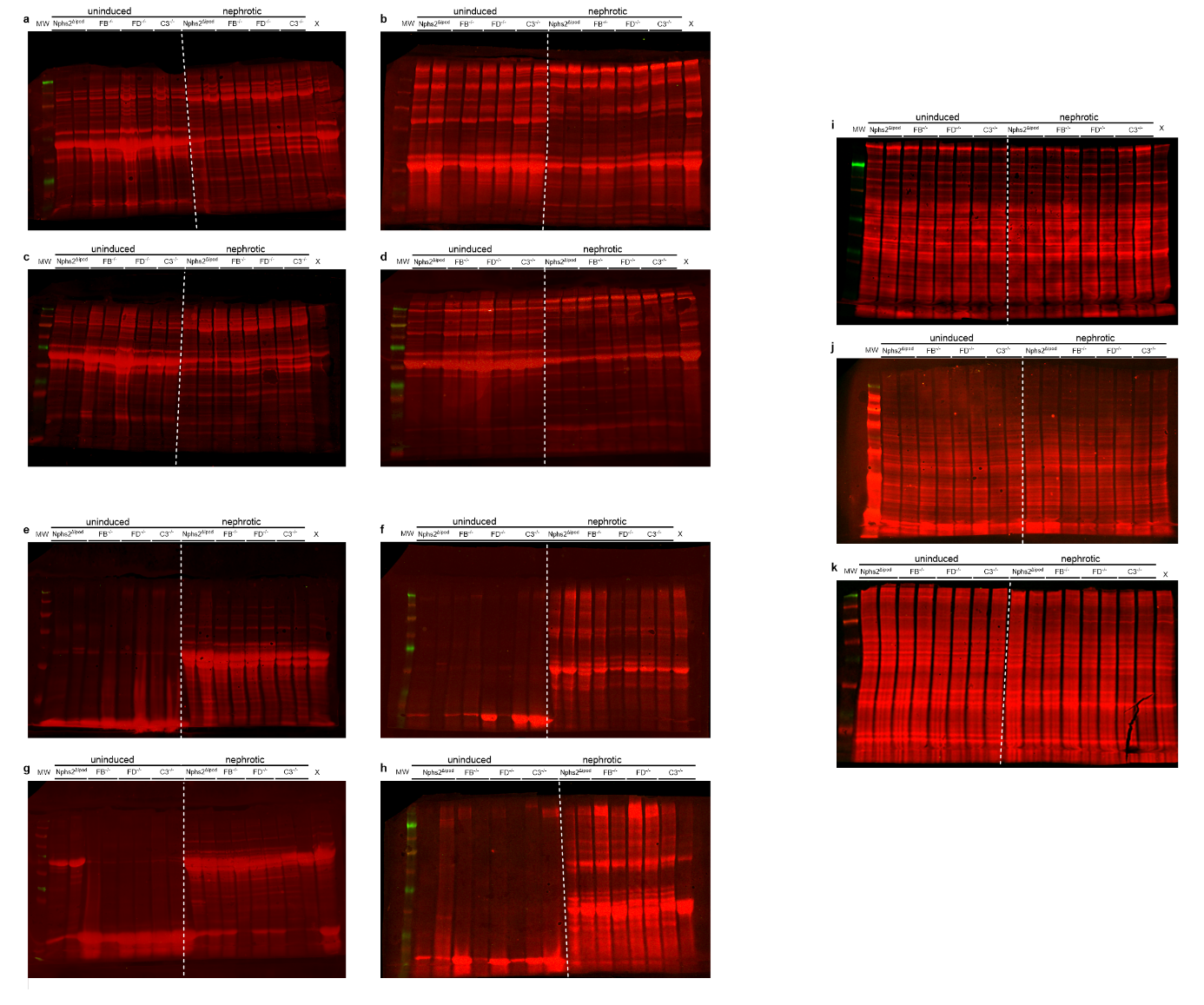


**Supplemental Figure 3. Food and fluid intake and urinary sodium in *Nphs2^Δipod^, Nphs2^Δipod^*Cfb^-/-^, Nphs2^Δipod^*Cfd^-/-^* and *Nphs2^Δipod^*C3^-/-^*** **mice before and after induction of nephrotic syndrome**

**a-c** Course of daily food intake

**d-f** Course of daily fluid intake

**h-j** Course of urinary sodium without correction on urinary creatinine

^#^ significant difference (p<0.05) between uninduced and nephrotic mice of the same genotype (ANOVA with Dunnetts multiple comparison test or Kruskal-Wallis with Dunn’s multiple comparison test), ^*^ significant difference (p<0.05) between genotypes and *Nphs2^Δipod^* (ANOVA with Dunnetts multiple comparison test or Kruskal-Wallis with Dunn’s multiple comparison test)


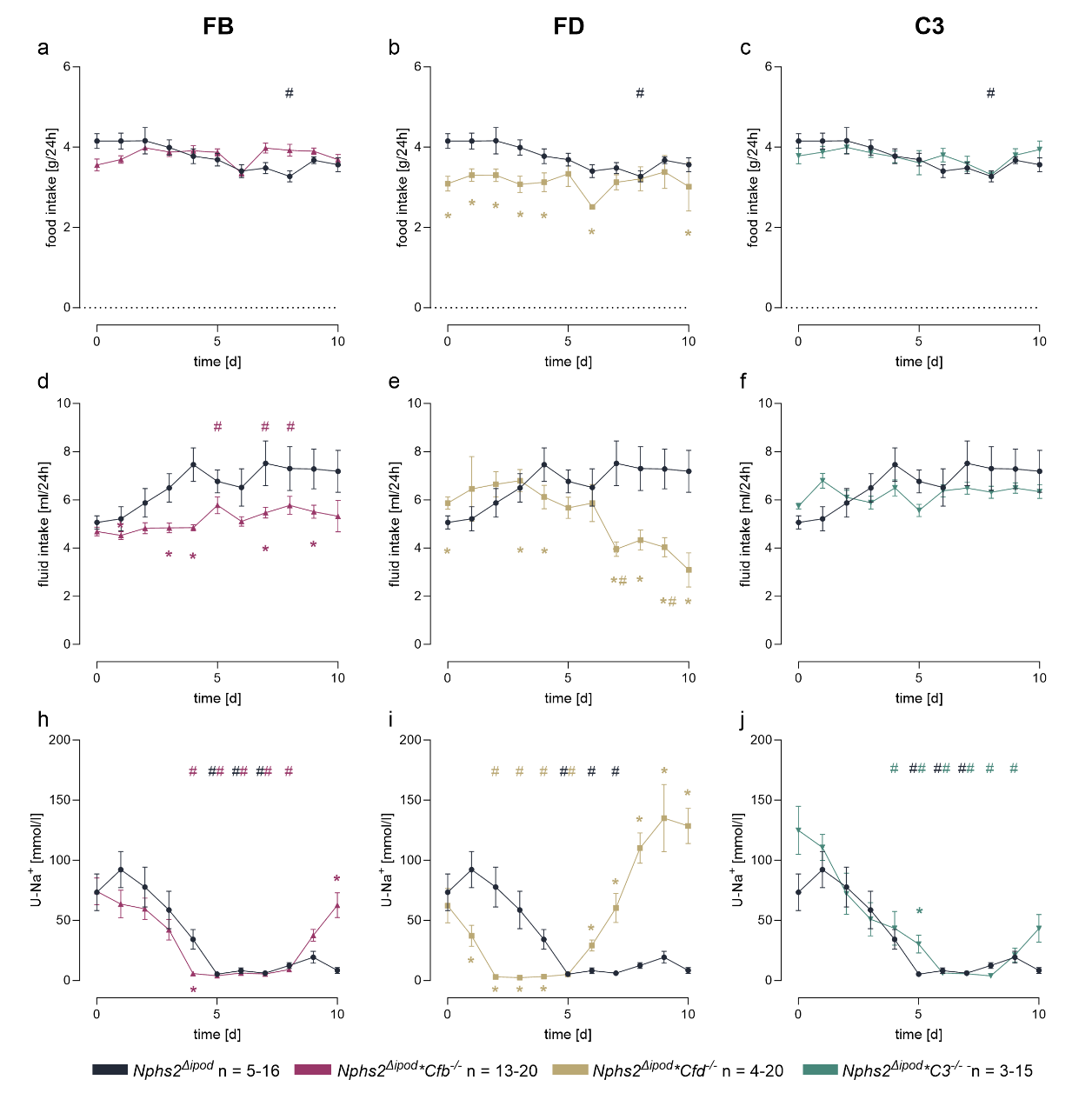


**References**

1. Mollet G, Ratelade J, Boyer O, Muda AO, Morisset L, Lavin TA, Kitzis D, Dallman MJ, Bugeon L, Hubner N (2009) Podocin inactivation in mature kidneys causes focal segmental glomerulosclerosis and nephrotic syndrome. J Am Soc Nephrol 20. doi:10.1681/asn.2009040379

2. Lin X, Suh JH, Go G, Miner JH (2014) Feasibility of repairing glomerular basement membrane defects in Alport syndrome. J Am Soc Nephrol 25:687-692. doi:10.1681/asn.2013070798

3. Perl AK, Wert SE, Nagy A, Lobe CG, Whitsett JA (2002) Early restriction of peripheral and proximal cell lineages during formation of the lung. Proceedings of the National Academy of Sciences of the United States of America 99:10482-10487. doi:10.1073/pnas.152238499

4. Circolo A, Garnier G, Fukuda W, Wang X, Hidvegi T, Szalai AJ, Briles DE, Volanakis JE, Wetsel RA, Colten HR (1999) Genetic disruption of the murine complement C3 promoter region generates deficient mice with extrahepatic expression of C3 mRNA. Immunopharmacology 42:135-149. doi:10.1016/s0162-3109(99)00021-1

5. Matsumoto M, Fukuda W, Circolo A, Goellner J, Strauss-Schoenberger J, Wang X, Fujita S, Hidvegi T, Chaplin DD, Colten HR (1997) Abrogation of the alternative complement pathway by targeted deletion of murine factor B. Proceedings of the National Academy of Sciences of the United States of America 94:8720-8725. doi:10.1073/pnas.94.16.8720

6. Xu Y, Ma M, Ippolito GC, Schroeder HW, Jr., Carroll MC, Volanakis JE (2001) Complement activation in factor D-deficient mice. Proceedings of the National Academy of Sciences of the United States of America 98:14577-14582. doi:10.1073/pnas.261428398

7. Masilamani S, Kim GH, Mitchell C, Wade JB, Knepper MA (1999) Aldosterone-mediated regulation of ENaC alpha, beta, and gamma subunit proteins in rat kidney. J Clin Invest 104. doi:10.1172/jci7840

8. Wu T, Dejanovic B, Gandham VD, Gogineni A, Edmonds R, Schauer S, Srinivasan K, Huntley MA, Wang Y, Wang TM, Hedehus M, Barck KH, Stark M, Ngu H, Foreman O, Meilandt WJ, Elstrott J, Chang MC, Hansen DV, Carano RAD, Sheng M, Hanson JE (2019) Complement C3 Is Activated in Human AD Brain and Is Required for Neurodegeneration in Mouse Models of Amyloidosis and Tauopathy. Cell Rep 28:2111-2123.e2116. doi:10.1016/j.celrep.2019.07.060

9. Pinto AK, Ramos HJ, Wu X, Aggarwal S, Shrestha B, Gorman M, Kim KY, Suthar MS, Atkinson JP, Gale Jr M, Diamond MS (2014) Deficient IFN Signaling by Myeloid Cells Leads to MAVS-Dependent Virus-Induced Sepsis. PLOS Pathogens 10:e1004086. doi:10.1371/journal.ppat.1004086

10. Williams JA, Stampoulis D, Gunter CE, Greenwood J, Adamson P, Moss SE (2016) Regulation of C3 Activation by the Alternative Complement Pathway in the Mouse Retina. PLoS One 11:e0161898. doi:10.1371/journal.pone.0161898

11. Vogel CW, Fritzinger DC (2010) Cobra venom factor: Structure, function, and humanization for therapeutic complement depletion. Toxicon 56:1198-1222. doi:10.1016/j.toxicon.2010.04.007
